# Plasma amyloid-beta levels correlated with impaired hepatic functions: An adjuvant biomarker for the diagnosis of biliary atresia

**DOI:** 10.3389/fsurg.2022.931637

**Published:** 2022-09-05

**Authors:** Hongyu Lyu, Yongqin Ye, Vincent Chi Hang Lui, Weifang Wu, Patrick Ho Yu Chung, Kenneth Kak Yuen Wong, Hung-Wing Li, Man Shing Wong, Paul Kwong Hang Tam, Bin Wang

**Affiliations:** ^1^ Graduate School, China Medical University, Shenyang, China; ^2^ Department of General Surgery, Shenzhen Children’s Hospital, Shenzhen, China; ^3^ Faculty of Medicine, Macau University of Science and Technology, Avenida Wai Long, Taipa, Macau SAR, China; ^4^ Department of Surgery, School of Clinical Medicine, The University of Hong Kong, Pokfulam, Hong Kong SAR, China; ^5^ Dr. Li Dak-Sum Research Centre, The University of Hong Kong, Pokfulam, Hong Kong SAR, China; ^6^ Medical College, Shantou University Medical College, Shantou, China; ^7^ Department of Surgery, University of Hong Kong-Shenzhen Hospital, Shenzhen, China; ^8^ Department of Chemistry, The Chinese University of Hong Kong, Shatin, Hong Kong SAR, China; ^9^ Department of Chemistry, Hong Kong Baptist University, Kowloon Tong, Hong Kong SAR, China

**Keywords:** biliary atresia, amyloid-beta, hepatic function, biomarker, diagnosis

## Abstract

**Background:**

Biliary atresia (BA) is an infantile fibro-obstructive cholestatic disease with poor prognosis. An early diagnosis and timely Kasai portoenterostomy (KPE) improve clinical outcomes. Aggregation of amyloid-beta (Aβ) around hepatic bile ducts has been discovered as a factor for BA pathogenesis, yet whether plasma Aβ levels correlate with hepatic dysfunctions and could be a biomarker for BA remains unknown.

**Method:**

Plasma samples of 11 BA and 24 controls were collected for liver function test, Aβ40 and Aβ42 measurement by enzyme-linked immunosorbent assay (ELISA). Pearson's chi-squared test or Mann–Whitney U test was performed to assess differences between groups. Correlation between Aβ42/Aβ40 and liver function parameters was performed using Pearson analysis. The area under the receiver-operative characteristic (ROC) curve (area under curve; AUC) was measured to evaluate the diagnostic power of Aβ42/Aβ40 for BA. Diagnostic enhancement was further evaluated by binary regression ROC analysis of Aβ42/Aβ40 combined with other hepatic function parameters.

**Results:**

Plasma Aβ42/Aβ40 was elevated in BA patients. Aβ42 displayed a weak positive correlation with γ-glutamyl transpeptidase (GGT) (Pearson’s correlation = 0.349), while there was no correlation for Aβ40 with hepatic functions. Aβ42/Aβ40 was moderately correlated with GGT, total bile acid (TBA), direct bilirubin (DBIL) (Pearson’s correlation = 0.533, 0.475, 0.480), and weakly correlated with total bilirubin (TBIL) (Pearson’s correlation = 0.337). Aβ42/Aβ40 showed an acceptable predictive power for cholestasis [AUC = 0.746 (95% CI: 0.552–0.941), *p *< 0.05]. Diagnostic powers of Aβ42/Aβ40 together with hepatic function parameters for cholestasis were markedly improved compared to any indicator alone. Neither Aβ42/Aβ40 nor hepatic function parameters displayed sufficient power in discriminating BA from choledochal cysts (CC); however, combinations of Aβ42/Aβ40 + GGT along with any other hepatic function parameters could differentiate BA from CC-cholestasis (AUC = 1.000, *p *< 0.05) with a cut-off value as 0.02371, −0.28387, −0.34583, 0.06224, 0.01040, 0.06808, and 0.05898, respectively.

**Conclusion:**

Aβ42/Aβ40 is a good indicator for cholestasis, but alone is insufficient for a distinction of BA from non-BA. However, Aβ42/Aβ40 combined with GGT and one other hepatic function parameter displayed a high predictive power as a screening test for jaundiced neonates who are more likely to be BA, enabling them to early intraoperative cholangiography for BA confirmation and KPE to improve surgical outcomes. However, a multi-centers validation is needed before introduction into daily clinical practice.

## Introduction

Biliary atresia (BA) is a progressive fibrosclerosing disease of the biliary tract, resulting in obstructive bile flow, cholestasis, and jaundice in young infants. BA affects all ethnicities with a noticeably higher incidence in the Asia–Pacific region (5–20:100,000 live-births) (1, 2). If untreated, patients develop progressive hepatic fibrosis leading to cirrhosis, portal hypertension, liver failure, and death by the age of two (1, 3). Kasai portoenterostomy (KPE) is the most widely accepted primary treatment. A timely KPE can potentially restore bile flow in 30%–80% of BA patients, but complications occur in many patients (4). It is estimated that up to 56%–74% of post-KPE BA patients at 10 years of age require liver transplantation (LT) (5).

Despite significant advances in BA management, clinicians face major challenges in establishing an early diagnosis for BA and predicting post-KPE outcomes. Early diagnosis is important because early KPE correlates with the best chances of good operative outcomes, delaying or even avoiding the need for LT (3). Currently, preoperative percutaneous liver biopsy has a higher specificity and sensitivity in early diagnosis of BA (6); however, an accurate, confirming diagnosis can only be established by surgical exploration and intraoperative cholangiography (7–9). Biomarkers are urgently needed for BA diagnosis. Plasma matrix metalloproteinase-7 (MMP7) (10–12) and γ-glutamyl transpeptidase (GGT) (13–16) have been suggested as promising tools in the diagnosis of BA; however, these studies have limitations such as different quantitation methods and different cut-off values were used. Furthermore, diagnostic accuracies of GGT for BA vary considerably in different age groups (16).

The amyloid precursor protein (APP) is pivotal in the pathophysiology of Alzheimer's disease (AD) since its abnormal cleavage by β-secretase and γ-secretase generates the hydrophobic amyloid-beta (Aβ) peptides including the two major Aβ peptides amyloid-beta 40 (Aβ40) and amyloid-beta 42 (Aβ42), which aggregate into neurotoxic amyloid plaques in the brain tissues, one of the key pathological hallmarks of AD [for a review, see (17)]. Aggregation of the Aβ peptides into amyloids is conceived as the pathogenic trigger of a cascade leading to tau accumulation into neurofibrillary tangles, neuronal loss, and clinical dementia in AD. Plasma Aβ42 correlates with cerebrospinal fluid Aβ42 in AD patients, and blood Aβ42 has been proposed as an alternative biomarker for AD (18–20).

Using human and mouse liver organoid and transcriptomics, we found (i) human and mouse BA liver organoids exhibited aberrant morphology, disturbed apical-basal organization, defective cholangiocyte development and altered Aβ-related gene expression; (ii) Aβ peptide deposition in bile ducts of BA livers; and (iii) Aβ induced the aberrant morphology in control organoids (21). The aberrant organoid morphology and periductal Aβ deposition are novel pathobiological and diagnostic features of BA. Our data identified Aβ deposition, the main pathological feature of AD and cerebral amyloid angiopathy, around BA bile ducts, suggesting that BA could be grouped under amyloid diseases. Plasma Aβ levels associate with hepatic functions in adults with liver cirrhosis (22). However, whether or not plasma Aβ levels correlate with hepatic functions in neonates and could be a non-invasive biomarker for BA is not known.

In the current study, we performed statistical and correlation analysis of plasma levels of Aβ40, Aβ42, and Aβ42/Aβ40 in BA and non-BA neonates to determine if plasma levels of Aβ peptides correlate with hepatic functions and can be used as an adjuvant biomarker to enhance the diagnostic accuracy for BA.

## Materials and methods

### Patients

This study was conducted prospectively at Shenzhen Children's Hospital, China, based on a protocol developed by the Hong Kong-Macau research team. Infants diagnosed with BA (*N* = 11) were enrolled from November 2021 to February 2022. The non-BA control group subjects included infants who suffered from choledochal cysts (CC; *N* = 5) as well as those patients (*N* = 19) with conditions unrelated to the liver. Intraoperative cholangiography and histologic examination confirmed the diagnosis of BA. The study was approved by the ethical committee of Shenzhen Children's Hospital and informed consent was obtained from all guardians or legal representatives of patients. Patients' information was tabulated and shown in Supplementary Table S1.

### Collection and preparation of plasma

Peripheral blood was collected into a vacuum tube containing EDTA at the time of KPE (BA patients) or at admission (non-BA subjects) and centrifuged (1,600 rpm for 10 min at 4 °C) to collect plasma for clinical laboratory tests and storage at −80 °C until Aβ peptides level quantitation. The clinical laboratory tests included the alanine aminotransferase (ALT), aspartate aminotransferase (AST), GGT, total bile acids (TBAs), total bilirubin (TBIL), direct bilirubin (DBIL), and indirect bilirubin (IBIL) levels.

### Plasma Aβ40 and Aβ42 measurement

Plasma Aβ40 and Aβ42 levels were measured using enzyme-linked immunosorbent assay (ELISA) kits [no. 27,718 human amyloid β (1–40) (FL) Assay Kit and no. 27,719 human amyloid β (1–42) (FL) Assay Kit, IBL, Gunma, Japan] according to the manufacturer's instructions. In brief, 100 µl of undiluted plasma samples were added to each well of the assay plate and incubated overnight at 4 °C. After washing, 100 µl of labeled antibody solution was added to each well, and the plate was incubated for 1 h at 4 °C. After washing, color was developed by incubation with 100 µl of chromogen at room temperature in dark, and the reactions were stopped by the addition of stop solution. The absorbance was measured at 450 nm using a plate reader (Infinite F50, TECAN), and the concentrations of (pg/ml) were calculated with reference to standard curves. Aβ42/Aβ40 ratios were multiplied by 100 and log_2_-transformed before being subjected for statistical analysis.

### Statistical analysis

Variables were presented as mean ± SEM if normally distributed, and otherwise as Median (Q1, Q3) values. Continuous data if normally distributed were compared using analysis of variance (ANOVA). The Pearson's chi-squared test was performed to assess differences between groups. The Mann–Whitney *U* test was performed for the continuous variables which were non-normally distribution. The correlation between Aβ42/Aβ40 and each of the liver function biochemical parameters was performed using Pearson (0.2–0.4: weak correlation; 0.4–0.7: moderate correlation; >0.7: strong correlation). The area under the receiver-operative characteristic (ROC) curve (area under curve; AUC) was calculated for Aβ42/Aβ40. For Aβ42/Aβ40 combined with positively correlated parameters of hepatic functions to predict BA, a binary regression for each independent variable was performed first, then a Logit(*P*) equation was derived, followed by ROC analysis. The sensitivity, specificity, Youden index of diagnostic test were calculated by discriminant analysis. All statistical analyses were performed with SPSS 26. A *p*-value <0.05 was considered statistically significant.

## Results

### Demographic characteristics of subjects

**Figure 1:**
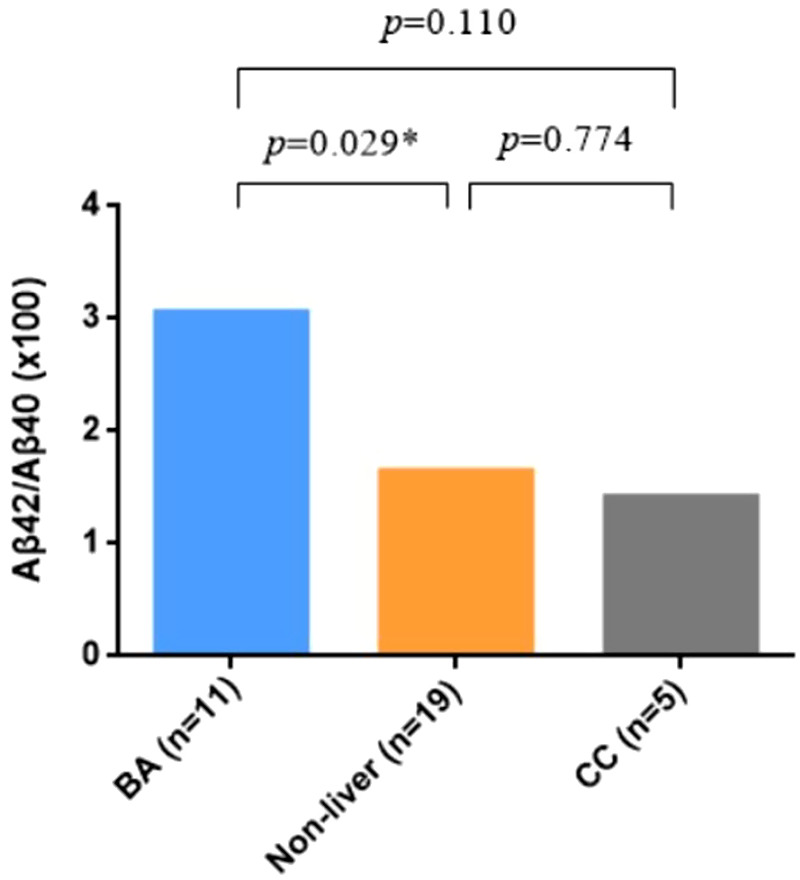
Expression level of plasma Aβ42/Aβ40 ratio in biliary atresia (BA) vs. non-BA. Mann–Whitney test was performed for Aβ42/Aβ40 ratio in different groups, showing that it was significantly elevated in BA patients.

The demographic data of BA and non-BA patients are shown in Table 1. The age of BA group was younger that the non-BA group, but there was no difference in gender between the two groups. Significantly higher levels of TBA, TBIL, DBIL, IBIL, ALT, AST, GGT were detected in BA plasma, which indicated impaired hepatic functions in BA patients. Aβ42 and Aβ40 were not significantly different, but Aβ42/Aβ40 ratio was significantly elevated in BA as compared to the non-BA group (Figure 1).

**Table 1:** Demographics of biliary atresia and non-biliary atresia patients.

	BA (*N* = 11)	Non-liver (*N* = 19)	CC (*N* = 5)	*p*-Value^a^	*p*-Value^b^	*p*-Value^c^
Age (month)	1.7 ± 0.2	3.9 ± 0.5	19.4 ± 6.0	0.001	0.118	0.170
Gender (male)	5	10	5	0.5	0.106	0.047
Aβ42 (pg/ml)	7.7 (5.5, 9.3)	4.5 (2.3, 9.3)	4.7 (3.1, 7.3)	0.171	0.086	0.956
Aβ40 (pg/ml)	286.9 (201.4, 375.6)	303.7 (240.5, 413.7)	355.6 (198.6, 405.2)	0.657	0.582	0.944
Aβ42/Aβ40 (×100)	3.1 (1.6, 4.0)	1.7 (1.3, 2.3)	1.4 (1.4, 2.1)	0.029	0.110	0.774
ALT IU/L (8–71)	124.0 (76.0, 249.0)	27.0 (20.0, 31.0)	223.0 (13.5, 294.0)	0.003	0.394	0.188
AST IU/L (21–80)	173.0 (100.0, 225.0)	42.0 (34.0, 51.0)	58.0 (28.5, 208.0)	0.001	0.169	0.318
GGT IU/L (29–80)	300.0 (168.0, 1011.0)	21.0 (16.0, 34.0)	375.0 (155.5, 621.0)	<0.001	0.893	0.001
TBA µmol/L (0.5–10)	122.2 (99.2, 188.3)	12.5 (5.5, 26.1)	34.0 (6.0, 105.7)	<0.001	0.060	0.274
TBIL µmol/L (0–17.1)	148 (114.9, 183.4)	7.9 (6.8, 14.5)	13.5 (9.0, 179.2)	<0.001	0.178	0.105
DBIL µmol/L (0–6.8)	91.8 (80, 111.1)	2.9 (2.2, 5.6)	7.0 (3.1, 78.1)	<0.001	0.065	0.142
IBIL µmol/L (2–17)	47.7 (24.5, 56.2)	5.3 (3.2, 9.4)	9.6 (4.4, 101.1)	<0.001	0.222	0.161

Values in parenthesis indicated normal range of hepatic function indicators. Levels of Aβ peptides and hepatic function indicators in patients were shown as Median (Q1, Q3). Age was shown as mean ± SEM.

BA, biliary atresia; CC, choledochal cysts; ALT, alanine aminotransferase; AST, aspartate aminotransferase; GGT, γ-glutamyl transpeptidase; TBA, total bile acid; TBIL, total bilirubin; DBIL, direct bilirubin; IBIL, indirect bilirubin.

^a^ BA vs. non-liver.

^b^ BA vs. CC.

^c^ Non-liver vs. CC.

### Positive correlation of plasma Aβ42/Aβ40 with hepatic functions

Next, Pearson correlation analysis was performed to determine if plasma Aβ42, Aβ40, and Aβ42/Aβ40 ratios correlated with hepatic functions in neonates. As shown in Table 2, plasma Aβ42 displayed a weak positive correlation with GGT, but no positive correlation with hepatic functions was detected for Aβ40. In contrast, Pearson correlation analysis revealed a statistically significant moderate positive correlation between Aβ42/Aβ40 and GGT, TBA, DBIL, and weak positive correlation between Aβ42/Aβ40 and TBIL (Table 2). Among children with non-liver related diseases, no significant correlation was identified between plasma Aβ42, plasma Aβ40, and plasma Aβ42/Aβ40 ratio and age, which suggested that age factor did not have much effect on their plasma levels (Supplementary Table S2).

**Table 2:** Pearson correlation analysis of variables.

		Aβ42	Aβ40	Aβ42/Aβ40	ALT	AST	GGT	TBA	TBIL	DBIL	IBIL
Aβ42	Pearson correlation	1	0.544**	0.233	−0.001	0.111	0.349*	0.157	0.276	0.241	0.211
	Sig. (two-tailed)		0.001	0.178	0.998	0.524	0.040	0.384	0.109	0.163	0.224
	*N*	35	35	35	35	35	35	33	35	35	35
Aβ40	Pearson correlation	0.544**	1	−0.312	−0.172	−0.128	−0.236	−0.230	−0.117	−0.190	−0.004
	Sig. (two-tailed)	0.001		0.068	0.324	0.462	0.172	0.199	0.504	0.274	0.983
	*N*	35	35	35	35	35	35	33	35	35	35
Aβ42/Aβ40	Pearson correlation	0.233	−0.312	1	0.283	0.272	0.533**	0.475**	0.337*	0.480**	0.078
	Sig. (two-tailed)	0.178	0.068		0.099	0.114	0.001	0.005	0.048	0.004	0.657
	*N*	35	35	35	35	35	35	33	35	35	35
ALT	Pearson correlation	−0.001	−0.172	0.283	1	0.869**	0.439**	0.604**	0.309	0.566**	−0.052
	Sig. (two-tailed)	0.998	0.324	0.099		0.000	0.008	0.000	0.071	0.000	0.767
	*N*	35	35	35	35	35	35	33	35	35	35
AST	Pearson correlation	0.111	−0.128	0.272	0.869**	1	0.437**	0.846**	0.509**	0.789**	0.055
	Sig. (two-tailed)	0.524	0.462	0.114	0.000		0.009	0.000	0.002	0.000	0.755
	*N*	35	35	35	35	35	35	33	35	35	35
GGT	Pearson correlation	0.349*	−0.236	0.533**	0.439**	0.437**	1	0.477**	0.690**	0.641**	0.491**
	Sig. (two-tailed)	0.040	0.172	0.001	0.008	0.009		0.005	0.000	0.000	0.003
	*N*	35	35	35	35	35	35	33	35	35	35
TBA	Pearson correlation	0.157	−0.230	0.475**	0.604**	0.846**	0.477**	1	0.648**	0.926**	0.146
	Sig. (two-tailed)	0.384	0.199	0.005	0.000	0.000	0.005		0.000	0.000	0.417
	*N*	33	33	33	33	33	33	33	33	33	33
TBIL	Pearson correlation	0.276	−0.117	0.337*	0.309	0.509**	0.690**	0.648**	1	0.812**	0.824**
	Sig. (two-tailed)	0.109	0.504	0.048	0.071	0.002	0.000	0.000		0.000	0.000
	*N*	35	35	35	35	35	35	33	35	35	35
DBIL	Pearson correlation	0.241	−0.190	0.480**	0.566**	0.789**	0.641**	0.926**	0.812**	1	0.339**
	Sig. (two-tailed)	0.163	0.274	0.004	0.000	0.000	0.000	0.000	0.000		0.046
	*N*	35	35	35	35	35	35	33	35	35	35
IBIL	Pearson correlation	0.211	−0.004	0.078	−0.052	0.055	0.491**	0.146	0.824**	0.339**	1
	Sig. (two-tailed)	0.224	0.983	0.657	0.767	0.755	0.003	0.417	0.000	0.046	
	*N*	35	35	35	35	35	35	33	35	35	35

ALT, alanine aminotransferase; AST, aspartate aminotransferase; GGT, γ-glutamyl transpeptidase; TBA, total bile acid; TBIL, total bilirubin; DBIL, direct bilirubin; IBIL, indirect bilirubin.

* Correlation is significant at the 0.05 level (two-tailed).

** Correlation is significant at the 0.01 level (two-tailed).

### Plasma Aβ42/Aβ40 for the diagnosis of cholestasis

**Figure 2:**
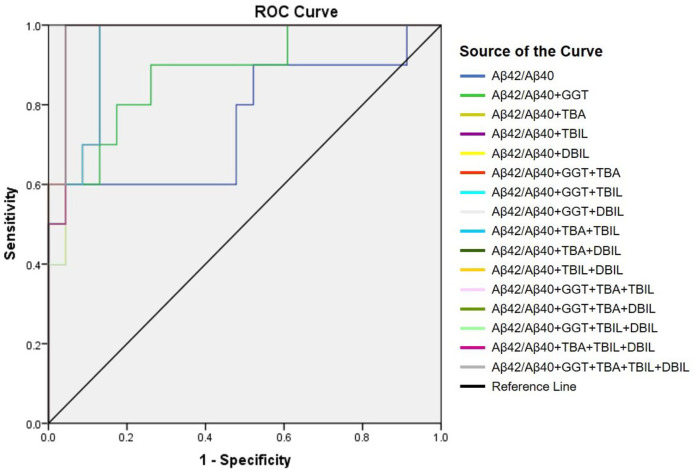
Receiver-operating characteristic (ROC) curve of Aβ42/Aβ40 alone and together with other hepatic function parameters.

Positive correlation of plasma Aβ42/Aβ40 with hepatic function parameters prompted us to examine if plasma Aβ peptides could be a biomarker for cholestasis. We performed ROC analysis to evaluate the efficacies of Aβ42, Aβ40, Aβ42/Aβ40, and hepatic function parameters in the diagnosis of cholestasis. Plasma Aβ42/Aβ40 had an acceptable predictive power for cholestasis [AUC = 0.746 (95% CI: 0.552–0.941), *p *< 0.05]; however, Aβ40 and Aβ42 did not show a significant predictive power for cholestasis (Table 3). Plasma ALT, AST, GGT, TBA, TBIL, DBIL, and IBIL had variable predictive power for cholestasis [AUC ranging from 0.746 to 0.962; (95% CI: 0.604–1.000), *p *< 0.05]. ROC analysis for Aβ42/Aβ40 alone and in combinations with different hepatic function parameters showed that the diagnostic powers of Aβ42/Aβ40 and each of the hepatic function parameters for cholestasis were markedly improved if combined (Table 3 and Figure 2).

**Table 3:** Area under curve of Aβ42, Aβ40, Aβ42/Aβ40, and hepatic function parameters for the diagnosis of cholestasis.

Test result variable(s)	Area	Std. error^a^	Asymptotic sig.^b^	Asymptotic 95% confidence interval	
				Lower bound	Upper bound
ALT	0.788	0.094	0.007	0.604	0.972
AST	0.833	0.084	0.002	0.669	0.997
GGT	0.898	0.053	0.000	0.795	1.000
TBA	0.928	0.051	0.000	0.828	1.000
TBIL	0.902	0.054	0.000	0.795	1.000
DBIL	0.962	0.037	0.000	0.889	1.000
IBIL	0.856	0.068	0.001	0.723	0.989
Aβ42	0.682	0.090	0.088	0.506	0.858
Aβ40	0.439	0.110	0.570	0.224	0.655
Aβ42/Aβ40	0.746	0.099	0.021	0.552	0.941
Aβ42/Aβ40 + GGT	0.875	0.063	0.000	0.751	0.999
Aβ42/Aβ40 + TBA	0.983	0.019	0.000	0.944	1.000
Aβ42/Aβ40 + TBIL	0.939	0.039	0.000	0.863	1.000
Aβ42/Aβ40 + DBIL	0.977	0.024	0.000	0.930	1.000
Aβ42/Aβ40 + GGT + TBA	0.983	0.019	0.000	0.944	1.000
Aβ42/Aβ40 + GGT + TBIL	0.939	0.039	0.000	0.863	1.000
Aβ42/Aβ40 + GGT + DBIL	0.977	0.024	0.000	0.930	1.000
Aβ42/Aβ40 + TBA + TBIL	0.978	0.023	0.000	0.933	1.000
Aβ42/Aβ40 + TBA + DBIL	0.978	0.023	0.000	0.933	1.000
Aβ42/Aβ40 + TBIL + DBIL	0.973	0.027	0.000	0.920	1.000
Aβ42/Aβ40 + GGT + TBA + TBIL	0.978	0.023	0.000	0.933	1.000
Aβ42/Aβ40 + GGT + TBA + DBIL	0.978	0.023	0.000	0.933	1.000
Aβ42/Aβ40 + GGT + TBIL + DBIL	0.973	0.027	0.000	0.920	1.000
Aβ42/Aβ40 + TBA + TBIL + DBIL	0.978	0.023	0.000	0.933	1.000
Aβ42/Aβ40 + GGT + TBA + TBIL + DBIL	0.983	0.019	0.000	0.944	1.000

ALT, alanine aminotransferase; AST, aspartate aminotransferase; GGT, γ-glutamyl transpeptidase; TBA, total bile acid; TBIL, total bilirubin; DBIL, direct bilirubin; IBIL, indirect bilirubin.

^a^ Under the nonparametric assumption.

^b^ Null hypothesis: true area = 0.5.

### Plasma Aβ42/Aβ40 together with hepatic function parameters for the diagnosis of biliary atresia

Neonatal pathological cholestasis can be caused by a number of disorders such as BA or non-BA cholestasis like CC, viral infections like cytomegalovirus (CMV), and metabolic liver diseases or genetic disorders like Alagille syndrome. Since BA was the most severe cholestatic disease, we next sought to test if Aβ42/Aβ40 and hepatic function parameters either alone or in combinations could well discriminate BA cholestasis from non-BA cholestasis by performing ROC analysis on the 11 BA patients and the two CC patients with cholestasis (patient nos. 12 and 16; Supplementary Table S1). Though Aβ42/Aβ40 was found to be a good indicator for cholestasis, it did not display sufficient power in differentiating BA cholestasis from CC-cholestasis, neither did any other hepatic function parameters (Tables 4, 5). However, when Aβ42/Aβ40 combined with GGT, a liver enzyme elevated in patients with biliary tract obstruction, it improved the efficiency to prone to BA (AUC = 0.955, *p *= 0.048). While combination of Aβ42/Aβ40 and GGT, and then with any one liver function parameters could further enhance the power to inform cholestasis patients to be BA or non-BA (AUC = 1.000, *p < *0.05) (Tables 4, 6, and Supplementary Table S3), which had higher power than the same combinations but without Aβ42/Aβ40 (Table 6), indicating the role of Aβ42/Aβ40 together with other parameters could be used as an adjuvant indicator to enhance the accuracy in distinguishing BA from non-BA.

**Table 4:** Area under curve of Aβ42/Aβ40 and hepatic function parameters either alone or in combinations for the diagnosis of biliary atresia.

Test result variable (s)	Area	Std. error^a^	Asymptotic sig^b^	Asymptotic 95% confidence Interval	
				Lower bound	Upper bound
GGT	0.364	0.151	0.554	0.068	0.659
Aβ42/Aβ40	0.545	0.150	0.844	0.251	0.840
Aβ42/Aβ40 + GGT	0.955	0.062	0.048	0.833	1.000
Aβ42/Aβ40 + GGT + TBA	1.000	0.000	0.032	1.000	1.000
Aβ42/Aβ40 + GGT + TBIL	1.000	0.000	0.030	1.000	1.000
Aβ42/Aβ40 + GGT + DBIL	1.000	0.000	0.030	1.000	1.000
Aβ42/Aβ40 + GGT + TBA + TBIL	1.000	0.000	0.032	1.000	1.000
Aβ42/Aβ40 + GGT + TBA + DBIL	1.000	0.000	0.032	1.000	1.000
Aβ42/Aβ40 + GGT + TBIL + DBIL	1.000	0.000	0.030	1.000	1.000
Aβ42/Aβ40 + GGT + TBA + TBIL + DBIL	1.000	0.000	0.032	1.000	1.000
ALT	0.636	0.221	0.554	0.204	1.000
AST	0.682	0.242	0.430	0.208	1.000
TBA	0.575	0.253	0.747	0.080	1.000
TBIL	0.227	0.126	0.236	0.000	0.474
DBIL	0.545	0.326	0.844	0.000	1.000
IBIL	0.182	0.123	0.167	0.000	0.424
Aβ42	0.682	0.242	0.430	0.208	1.000
Aβ40	0.545	0.273	0.844	0.011	1.000
Aβ42/Aβ40 + TBA	0.750	0.159	0.283	0.439	1.000
Aβ42/Aβ40 + TBIL	0.727	0.134	0.324	0.464	0.990
Aβ42/Aβ40 + DBIL	0.727	0.214	0.324	0.309	1.000
Aβ42/Aβ40 + TBA + TBIL	0.800	0.134	0.197	0.537	1.000
Aβ42/Aβ40 + TBA + DBIL	0.750	0.202	0.283	0.355	1.000
Aβ42/Aβ40 + TBIL + DBIL	0.773	0.185	0.236	0.410	1.000
Aβ42/Aβ40 + TBA + TBIL + DBIL	0.800	0.170	0.197	0.466	1.000

ALT, alanine aminotransferase; AST, aspartate aminotransferase; GGT, γ-glutamyl transpeptidase; TBA, total bile acid; TBIL, total bilirubin; DBIL, direct bilirubin; IBIL, indirect bilirubin.

^a^ Under the nonparametric assumption.

^b^ Null hypothesis: true area = 0.5.

**Table 5:** Sensitivity and specificity of Aβ42/Aβ40 and γ-glutamyl transpeptidase in combinations with any one hepatic function parameters in the diagnosis of biliary atresia vs. non-biliary atresia cholestasis.

Parameters	Cut-off value	Sensitivity (%)	Specificity (%)	Equation
Aβ42/Aβ40 + GGT + TBA	−0.34583	100.00	100.00	22.549 + 35.326 * Aβ42/Aβ40 − 0.298 * GGT − 0.332 * TBA
Aβ42/Aβ40 + GGT + TBIL	0.02371	100.00	100.00	−526.079 + 287.021 * Aβ42/Aβ40 − 2.250 * GGT + 1.555 * TBIL
Aβ42/Aβ40 + GGT + DBIL	−0.28387	100.00	100.00	83.795 + 98.348 *vAβ42/Aβ40 − 0.805 * GGT − 1.383 * DBIL
Aβ42/Aβ40 + GGT + TBA + TBIL	0.01040	100.00	100.00	−48.789 + 25.050 * Aβ42/Aβ40 − 0.396 * GGT − 0.453 * TBA + 0.974 * TBIL
Aβ42/Aβ40 + GGT + TBA + DBIL	0.06808	100.00	100.00	14.881 + 31.888 *vAβ42/Aβ40 − 0.267 * GGT − 0.343 * TBA + 0.106 * DBIL
Aβ42/Aβ40 + GGT + TBIL + DBIL	0.06224	100.00	100.00	−86.646 + 38.587 * Aβ42/Aβ40 − 0.585 * GGT + 1.631 * TBIL − 1.143 * DBIL
Aβ42/Aβ40 + GGT + TBA + TBIL + DBIL	0.05898	100.00	100.00	−43.153 + 24.039 * Aβ42/Aβ40 − 0.378 * GGT − 0.403 * TBA + 0.934 * TBIL − 0.092 * DBIL

GGT, γ-glutamyl transpeptidase; TBA, total bile acid; TBIL, total bilirubin; DBIL, direct bilirubin; IBIL, indirect bilirubin.

**Table 6:** Area under the curve of γ-glutamyl transpeptidase and other hepatic function parameters with/without Aβ42/Aβ40 for the diagnosis of biliary atresia.

Test result variable(s)		Area	Std. error^a^	Asymptotic sig.^b^	Asymptotic 95% confidence interval	
					Lower bound	Upper bound
GGT + TBA	Without Aβ42/Aβ40	0.600	0.239	0.667	0.132	1.000
	With Aβ42/Aβ40	1.000	0.000	0.032	1.000	1.000
GGT + TBIL	Without Aβ42/Aβ40	0.636	0.151	0.554	0.341	0.932
	With Aβ42/Aβ40	1.000	0.000	0.030	1.000	1.000
GGT + DBIL	Without Aβ42/Aβ40	0.545	0.326	0.844	0.000	1.000
	With Aβ42/Aβ40	1.000	0.000	0.030	1.000	1.000
GGT + TBA + TBIL	Without Aβ42/Aβ40	0.750	0.136	0.283	0.483	1.000
	With Aβ42/Aβ40	1.000	0.000	0.032	1.000	1.000
GGT + TBA + DBIL	Without Aβ42/Aβ40	0.550	0.323	0.830	0.000	1.000
	With Aβ42/Aβ40	1.000	0.000	0.032	1.000	1.000
GGT + TBIL + DBIL	Without Aβ42/Aβ40	0.636	0.221	0.554	0.204	1.000
	With Aβ42/Aβ40	1.000	0.000	0.030	1.000	1.000
GGT + TBA + TBIL + DBIL	Without Aβ42/Aβ40	0.700	0.232	0.390	0.245	1.000
	With Aβ42/Aβ40	1.000	0.000	0.032	1.000	1.000

GGT, γ-glutamyl transpeptidase; TBA, total bile acid; TBIL, total bilirubin; DBIL, direct bilirubin; IBIL, indirect bilirubin.

^a^ Under the nonparametric assumption.

^b^ Null hypothesis: true area = 0.5.

## Discussion

Neonatal jaundice is common, and up to two-thirds of all newborns develop this problem within the first 2 weeks of life (23). While most cases are physiological jaundice that are mild, transient, and self-limiting, more severe cases of pathological jaundice are not uncommon. Diseases including BA and CC are common pathological causes of neonatal cholestasis requiring timely intervention (24). However, BA is challenging to diagnose because many of the clinical and imaging features of this condition overlap with those of other causes of neonatal cholestasis. In this study, we found that plasma Aβ42/Aβ40 correlated with hepatic function parameters, and combinations of plasma Aβ42/Aβ40 with GGT, TBA, TBIL, or DBIL displayed sensitivity and specificity for the diagnosis of BA.

Plasma levels of liver enzymes (ALT, AST, and GGT) and various forms of bilirubin (TBIL, DBIL, and IBIL) are hepatic function indicators, which are used to assist the diagnosis of cholestatic liver disease liver. In line with the use of these hepatic function indicators in the diagnosis of cholestasis, we have observed that plasma levels of ALT, AST, GGT, TBIL, DBIL, and IBIL have predictive power for cholestasis. Pearson correlation analysis revealed positive correlations of plasma Aβ42/Aβ40 with GGT, TBA, DBIL, and TBIL. In line with the positive correlation between Aβ42/Aβ40 and hepatic function indicators, plasma Aβ42/Aβ40 also has a predictive power for cholestasis [AUC = 0.746 (95% CI: 0.552–0.941), *p *< 0.05]. More importantly, combinations of Aβ42/Aβ40 and hepatic function indicators markedly improved the diagnostic accuracies of Aβ42/Aβ40 and each of the hepatic function parameters for cholestasis, suggesting that plasma Aβ42/Aβ40 can be an additional biomarker enhancing the accuracies of those common hepatic function indicators for the diagnosis of cholestatic liver disease. There is no current evidence to show that Aβ42/Aβ40 varies with ages. In our study, we also found no correlation between age and plasma Aβ42, Aβ40, and Aβ42/Aβ40, indicating that age may not be a determining factor in their plasma levels.

Since neonatal cholestasis has many different causes including BA, the most severe and poorest prognosis, and other non-BA cholestasis such as viral infection including CMV and herpes viruses; metabolic liver diseases or genetic disorders such as alpha-1-antitrypsin deficiency and Alagille syndrome (25). Cholestasis caused by BA or non-BA diseases is difficult to differentiate clinically. Currently, liver function test parameters may have an indication for the diagnosis of BA. For example, serum DBIL ≥ 1.0 mg/dl had a better detection for BA with a sensitivity of 100% and specificity of 77.3% in infants aged from 3 to 60 days, but with a low positive predictive value (2.7%–5.4%) to distinguish BA from non-BA (26, 27). Hence it is important that new non-invasive biomarkers are discovered to allow early screening of BA from non-BA in jaundiced infants. Though Aβ42/Aβ40 was an indicator for liver dysfunction from our data, it was not sufficient on its own to distinguish BA from non-BA cholestasis. Similarly, the hepatic function parameters singly or collectively were insufficient for BA and non-BA differentiation. However, the combination of Aβ42/Aβ40 and GGT improved the efficiency of BA prediction compared to the use of either index alone. GGT, a liver enzyme elevated in patients with biliary tract obstruction, has been shown to have a contributory though not definitive role in the diagnosis of BA (16, 28). Addition of one of the other liver function parameters to the combination of Aβ42/Aβ40 and GGT could further enhance the power to inform the cause of cholestasis as BA or non-BA (AUC = 1.000, *p < *0.05). Taken all the above, our preliminary findings suggested that plasma Aβ42/Aβ40 could be a valuable adjuvant biomarker for BA diagnosis.

We acknowledge there are limitations in our study. As BA is a relatively rare disease, the sample size of our study is modest. Our plasma Aβ42/Aβ40 data are encouraging and corroborate with the tissue and organoids findings of our previous study (20). Nevertheless, the diagnostic accuracy and usefulness of Aβ42/Aβ40 as an adjuvant non-invasive biomarker in combination with other liver function parameters for BA needs to be further evaluated and validated in a separate larger patient cohort of BA, non-BA cholestatic liver diseases, and conditions unrelated to the liver in multiple centers before introduction into daily clinical practice.

In conclusion, by combining Aβ42/Aβ40 as an adjuvant biomarker with other liver function parameters, we improve the sensitivity and the specificity of BA diagnosis. As an early screening tool, this may allow the identification of jaundiced neonates who are more likely to be suffering from BA to undergo early surgical exploration and intraoperative cholangiography for BA confirmation and KPE, thus improving the surgical outcome.

## Data Availability

The original contributions presented in the study are included in the article/Supplementary Material, further inquiries can be directed to the corresponding authors.
