# Supplementary material for: Plasma amyloid-beta levels correlated with impaired hepatic functions: An adjuvant biomarker for the diagnosis of biliary atresia
Source: Front Surg. 2022 Sep 5;9:931637. doi: 10.3389/fsurg.2022.931637 (PMC9483031; doi:10.3389/fsurg.2022.931637)
Supplement: Supplementary file 1 [file Table_1_v1.docx]

**Supplementary Table 1.** Raw data of enrolled patients

| Patients No. | Gender | Age (mth) | Diagnosis | Aβ42, pg/ml | Aβ40, pg/ml | Aβ42/Aβ40 *100 | log_2_ (Aβ42/Aβ40 *100) | ALT IU/L (8-71) | AST IU/L (21-80) | GGT IU/L (29-80) | TBA  µmol/L (0.5-10) | TBIL µmol/L (0-17.1) | DBIL µmol/L (0-6.8) | IBIL µmol/L (2-17) |
| --- | --- | --- | --- | --- | --- | --- | --- | --- | --- | --- | --- | --- | --- | --- |
| 1 | female | 1.80 | BA | 7.7461 | 460.69 | 1.68 | 3.21 | 77 | 168 | 300 | 110.9 | 183.4 | 109 | 74.4 |
| 2 | male | 2.60 | BA | 10.654 | 100.93 | 10.56 | 1505.30 | 249 | 200 | 1177 | 233.9 | 217.3 | 163.8 | 53.5 |
| 3 | female | 0.83 | BA | 6.9016 | 225.53 | 3.06 | 8.34 | 14 | 39 | 1011 | 16.7 | 265.8 | 58.6 | 207.2 |
| 4 | female | 1.80 | BA | 5.4747 | 80.881 | 6.77 | 109.05 | 124 | 189 | 241 | 173.1 | 148 | 91.8 | 56.2 |
| 5 | female | 1.60 | BA | 4.6764 | 286.94 | 1.63 | 3.09 | 254 | 225 | 254 | 128.2 | 164.2 | 111.8 | 52.4 |
| 6 | male | 1.53 | BA | 8.9544 | 487.79 | 1.84 | 3.57 | 142 | 237 | 168 | 247.1 | 152.4 | 111.1 | 41.3 |
| 7 | female | 2.33 | BA | 12.804 | 321 | 3.99 | 15.88 | 100 | 137 | 1029 | 112.4 | 125.7 | 100.5 | 25.2 |
| 8 | female | 2.30 | BA | 6.0843 | 375.55 | 1.62 | 3.07 | 76 | 100 | 166 | N/A | 127.7 | 80 | 47.7 |
| 9 | male | 1.67 | BA | 9.2529 | 257.44 | 3.59 | 12.08 | 356 | 267 | 472 | 144.3 | 114.9 | 90.4 | 24.5 |
| 10 | male | 1.03 | BA | 3.6668 | 351.11 | 1.04 | 2.06 | 225 | 173 | 97 | 116.2 | 102.3 | 81.6 | 20.7 |
| 11 | male | 0.80 | BA | 8.0974 | 201.35 | 4.02 | 16.24 | 22 | 30 | 400 | 64 | 70 | 51.5 | 18.6 |
| 12 | female | 36 | CC | 1.8627 | 96.555 | 1.93 | 3.81 | 223 | 190 | 375 | 173.1 | 174.2 | 118.9 | 55.3 |
| 13 | female | 24 | CC | 5.7538 | 405.78 | 1.42 | 2.67 | 12 | 29 | 68 | 2.5 | 13.5 | 3.9 | 9.6 |
| 14 | female | 24 | CC | 4.6702 | 355.64 | 1.31 | 2.48 | 226 | 58 | 243 | 9.5 | 5.5 | 2.2 | 3.3 |
| 15 | female | 12 | CC | 4.2468 | 300.72 | 1.41 | 2.66 | 362 | 226 | 631 | 34 | 12.5 | 7 | 5.5 |
| 16 | female | 1 | CC | 8.8799 | 404.7 | 2.19 | 4.58 | 15 | 28 | 611 | 38.2 | 184.2 | 37.3 | 146.9 |
| 17 | male | 6 | Cryptorchidism | 10.22 | 409.57 | 2.50 | 5.64 | 25 | 38 | 8 | 10.7 | 7.9 | 2.6 | 5.3 |
| 18 | male | 6 | Cryptorchidism | 3.2269 | 204.85 | 1.58 | 2.98 | 27 | 55 | 9 | 45.5 | 6.8 | 3 | 3.8 |
| 19 | male | 2.53 | Indirect Inguinal Hernia | 3.0665 | 240.52 | 1.27 | 2.42 | 42 | 51 | 34 | 5.3 | 7.8 | 2.7 | 5.1 |
| 20 | female | 3 | Congenital Hip Dysplasia | 9.2561 | 304.85 | 3.04 | 8.20 | 45 | 63 | 36 | 13.5 | 15.6 | 6.2 | 9.4 |
| 21 | male | 3 | Congenital Clubfoot | 4.1499 | 303.72 | 1.37 | 2.58 | 21 | 28 | 20 | 5.8 | 5 | 1.8 | 3.2 |
| 22 | female | 2.8 | Umbilical Hernia | 9.5031 | 413.71 | 2.30 | 4.91 | 20 | 34 | 27 | 8 | 12.1 | 3.9 | 8.2 |
| 23 | female | 2.03 | Tethered Spinal Cord Syndrome | 3.785 | 291.15 | 1.30 | 2.46 | 30 | 44 | 63 | 25.8 | 21.4 | 8 | 13.4 |
| 24 | female | 4 | Hemangioma | 6.5211 | 326.86 | 2.00 | 3.99 | 21 | 47 | 17 | 27 | 7.3 | 2.2 | 5.1 |
| 25 | male | 2 | Hemangioma | 6.2372 | 423.68 | 1.47 | 2.77 | 20 | 31 | 120 | 21.1 | 91.6 | 12.3 | 79.3 |
| 26 | female | 1.43 | Hemangioma | 8.7471 | 316.15 | 2.77 | 6.81 | 21 | 38 | 73 | 11.7 | 173.8 | 9.6 | 164.2 |
| 27 | female | 7 | Congenital Muscular Torticollis | 11.903 | 470.48 | 2.53 | 5.78 | 29 | 42 | 16 | 1.8 | 14.5 | 5 | 9.5 |
| 28 | female | 3 | Indirect Inguinal Hernia | 1.3253 | 69.946 | 1.89 | 3.72 | 31 | 34 | 26 | 28.9 | 7.9 | 2.2 | 5.7 |
| 28 | male | 3 | Congenital Clubfoot | 12.041 | 546 | 2.21 | 4.61 | 51 | 53 | 18 | 21.5 | 12.9 | 5.6 | 7.3 |
| 30 | male | 6 | Gonarthritis | 1.7696 | 280.5 | 0.63 | 1.55 | 9 | 23 | 29 | N/A | 4 | 2.5 | 1.5 |
| 31 | male | 1.2 | Urinary Tract Infection | 2.3074 | 289.86 | 0.80 | 1.74 | 30 | 34 | 13 | 4.9 | 4.7 | 2.2 | 2.5 |
| 32 | female | 5 | Urinary Tract Infection | 1.578 | 115.59 | 1.37 | 2.58 | 29 | 50 | 21 | 13.2 | 6.9 | 3.9 | 3 |
| 33 | male | 10 | Acute Upper Respiratory  Tract Infection | 8.7989 | 421.99 | 2.09 | 4.24 | 18 | 45 | 9 | 2.4 | 3.5 | 1.8 | 1.7 |
| 34 | female | 3 | Hemangioma | 4.5414 | 275.16 | 1.65 | 3.14 | 68 | 68 | 25 | 35.2 | 10.4 | 2.9 | 7.5 |
| 35 | male | 4 | Urinary Tract Infection | 1.7066 | 181.15 | 0.94 | 1.92 | 18 | 29 | 20 | 5.6 | 7.7 | 2.5 | 5.2 |

N/A, Not Available
