# Supplementary material for: Plasma amyloid-beta levels correlated with impaired hepatic functions: An adjuvant biomarker for the diagnosis of biliary atresia
Source: Front Surg. 2022 Sep 5;9:931637. doi: 10.3389/fsurg.2022.931637 (PMC9483031; doi:10.3389/fsurg.2022.931637)
Supplement: Supplementary file 2 [file Table_2_v1.docx]

| **Supplementary Table 2.** Pearson correlation analysis of Aβ42, Aβ40, or Aβ42/Aβ40 with age among children with non-liver diseases | | | | | |
| --- | --- | --- | --- | --- | --- |
|  | | Age | Aβ42 | Aβ40 | Aβ42/Aβ40 |
| Age | Pearson Correlation | 1 | 0.205 | 0.160 | 0.070 |
|  | Sig. (2-tailed) |  | 0.399 | 0.513 | 0.777 |
|  | N | 19 | 19 | 19 | 19 |
| Aβ42 | Pearson Correlation | 0.205 | 1 | 0.859^a^ | 0.789^a^ |
|  | Sig. (2-tailed) | 0.399 |  | 0.000 | 0.000 |
|  | N | 19 | 19 | 19 | 19 |
| Aβ40 | Pearson Correlation | 0.160 | 0.859^a^ | 1 | 0.403 |
|  | Sig. (2-tailed) | 0.513 | 0.000 |  | 0.087 |
|  | N | 19 | 19 | 19 | 19 |
| Aβ42/Aβ40 | Pearson Correlation | 0.070 | 0.789^a^ | 0.403 | 1 |
|  | Sig. (2-tailed) | 0.777 | 0.000 | 0.087 |  |
|  | N | 19 | 19 | 19 | 19 |
| a. Correlation is significant at the 0.01 level (2-tailed). | | | | | |
