# Supplementary material for: Plasma amyloid-beta levels correlated with impaired hepatic functions: An adjuvant biomarker for the diagnosis of biliary atresia
Source: Front Surg. 2022 Sep 5;9:931637. doi: 10.3389/fsurg.2022.931637 (PMC9483031; doi:10.3389/fsurg.2022.931637)
Supplement: Supplementary file 3 [file Table_3_v1.docx]

**Supplementary Table 3.** Sensitivity and specificity of Aβ42/Aβ40 in combinations with hepatic function parameters other than GGT in the diagnosis of BA vs. non-BA cholestasis.

| Parameters | Cut-off Value | Sensitivity | Specificity | Equation |
| --- | --- | --- | --- | --- |
| Aβ42/Aβ40+GGT | 1.69120 | 90.91% | 100.00% | -10.995+9.876*Aβ42/40-0.068*GGT |
| Aβ42/Aβ40+TBA | 1.59824 | 60.00% | 100.00% | -1.101+0.276*Aβ42/40+0.008*TBA |
| Aβ42/Aβ40+TBIL | 1.18255 | 72.73% | 100.00% | 3.388+0.450*Aβ42/40-0.024*TBIL |
| Aβ42/Aβ40+DBIL | 0.31249 | 100.00% | 50.00% | -2.027+0.257*Aβ42/40+0.025*DBIL |
| Aβ42/Aβ40+TBA+TBIL | 1.16156 | 70.00% | 100.00% | 1.191+0.403*Aβ42/40+0.005*TBA-0.014*TBIL |
| Aβ42/Aβ40+TBA+DBIL | 2.55745 | 50.00% | 100.00% | -2.854+0.300*Aβ42/40-0.004*TBA+0.035*DBIL |
| Aβ42/Aβ40+TBIL+DBIL | 2.18245 | 54.55% | 100.00% | 1.085+1.185*Aβ42/40-0.044*TBIL+0.034*DBIL |
| Aβ42/Aβ40+TBA+TBIL+DBIL | 1.95671 | 60.00% | 100.00% | 2.225+1.673*Aβ42/40-0.022*TBA-0.071*TBIL+0.078*DBIL |
